# Axillary to Superior Mesenteric Artery Bypass as a Viable Solution for Chronic Mesenteric Ischemia

**DOI:** 10.7759/cureus.75524

**Published:** 2024-12-11

**Authors:** Lanah Almatroud, Isabel Billiar, Bart Chess, Mahmoud Elrakhawy

**Affiliations:** 1 College of Human Medicine, Michigan State University, East Lansing, USA; 2 Vascular Surgery, University of Pittsburgh Medical Center, Pittsburgh, USA; 3 Division of Vascular Surgery, Allegheny Health Network, Pittsburgh, USA; 4 Vascular Surgery, McGinnis Thoracic &amp; Cardiovascular Associates, Pittsburgh, USA; 5 Vascular Surgery, Carle Foundation Hospital, Urbana, USA

**Keywords:** aortic disease, atherosclerotic disease, chronic abdominal pain, chronic mesenteric ischemia (cmi), general and vascular surgery, non occlusive mesenteric ischemia, postoperative outcomes, surgical revascularization

## Abstract

Chronic mesenteric ischemia (CMI) is a progressive condition that primarily affects the elderly, causing chronic abdominal pain and malnutrition. Timely treatment is essential to prevent further deconditioning or bowel ischemia. Surgical repair options include both endovascular and open procedures. We present the case of a patient with CMI and diffuse atherosclerotic disease of the aorta, treated with a left axillary-to-superior mesenteric artery (SMA) bypass. This case highlights axillary-to-SMA bypass as a novel and effective solution for CMI in patients with complex vascular anatomy and diffuse vascular disease, providing a viable alternative when traditional methods are contraindicated. Following the procedure, the patient experienced resolution of abdominal symptoms, and a six-month abdominal CT scan confirmed a widely patent bypass.

## Introduction

Chronic mesenteric ischemia (CMI) is a progressive atherosclerotic disease that typically affects individuals over 65 years of age, predominantly affecting females. Major risk factors include smoking, hypertension, hypercholesterolemia, and a family history of atherosclerotic disease [1]. CMI generally develops when at least two of the three mesenteric vessels - commonly the celiac and superior mesenteric (SMA) arteries - experience clinically significant stenosis, often exceeding 70% [2]. The classic presentation includes postprandial abdominal pain beginning 30 minutes after a meal and lasting approximately 45 minutes. Over time, this progresses to chronic, dull abdominal pain and, if untreated, may result in catastrophic outcomes [3,4]. Patients may also experience “food fear,” leading to weight loss and malnutrition, which complicates tissue healing after open revascularization due to inadequate nutritional reserves.

Current treatments include both surgical and endovascular revascularization options, alongside optimization of modifiable risk factors [4,5]. Open mesenteric bypass has traditionally been the cornerstone of surgical reconstruction, with techniques such as anterograde or retrograde bypass grafting, transaortic endarterectomy, and direct mesenteric artery reimplantation. However, recent advances in endovascular therapy have led to angioplasty and stenting becoming the first-line treatment, particularly for patients deemed high-risk for open reconstruction [4]. Despite these advancements, patients with severe aortoiliac calcific disease and complex anatomy often face limited options.

This case adds to the growing body of evidence supporting axillary-to-SMA bypass as a feasible approach for these challenging scenarios. In this report, we describe the innovative use of a left axillary-to-SMA bypass graft for the treatment of CMI in a patient with diffuse aortoiliac calcific disease, offering a unique perspective on treating anatomical complexity and clinical challenges.

## Case presentation

**Figure 1:**
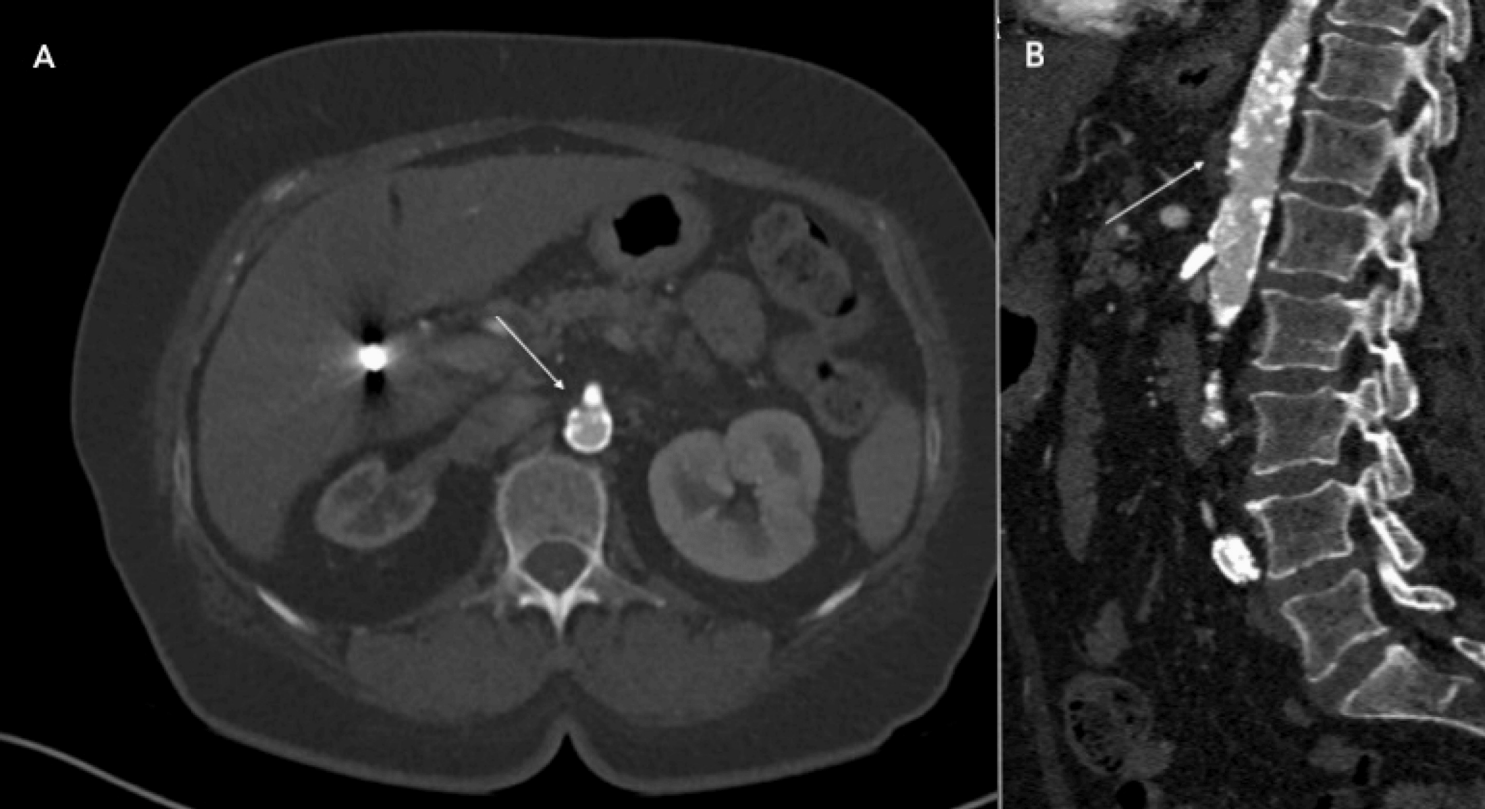
Superior mesenteric artery (SMA) occlusion on computed tomography angiogram (CTA). (A) Axial image showing circumferential calcification of the aorta (white arrow) with occlusive plaque at the ostium of the SMA. (B) Sagittal view of the aorta and SMA origin, demonstrating diffuse luminal calcifications of the aorta (white arrow) and a long occlusion of the origin and proximal SMA.

**Figure 2:**
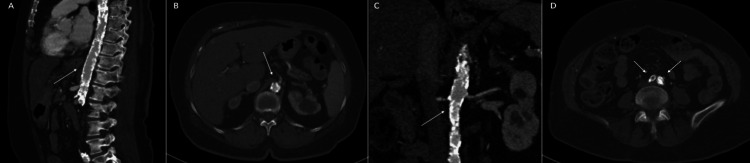
Pre-operative computed tomography angiogram (CTA) images of the aorta. (A) Sagittal view showing dense circumferential calcifications of the thoracic and abdominal aorta with areas of luminal compromise (white arrow). (B) Coronal view revealing similar calcifications and luminal narrowing (white arrow). (C) Severe calcific disease of the supra-celiac aorta (white arrow). (D) Significant narrowing of the aortic bifurcation and common iliac arteries (white arrow).

The patient is a 71-year-old woman with a medical history significant for severe peripheral arterial disease, chronic obstructive pulmonary disease (COPD) on home oxygen, and chronic stage III kidney disease. Past vascular interventions included a left carotid endarterectomy and bilateral iliac stent placement. She initially presented to the vascular surgery office with lifestyle-limiting bilateral lower extremity claudication. The patient reported severe food-related fear, limiting herself to one meal a day and experiencing significant weight loss. Larger meals were associated with intractable abdominal pain, nausea, and vomiting. Her workup included a computed tomography angiogram (CTA) of the chest, abdomen, and pelvis, alongside arteriography. These studies demonstrated obliterative calcific disease of the thoracoabdominal aorta, near-occlusive calcific plaque at the SMA ostium, and high-grade stenosis of the celiac axis (Figure 1), and a small inferior mesenteric artery (IMA) with segmental high-grade stenosis. Pre-operative CTA images also revealed dense circumferential calcifications of the thoracic and abdominal aorta with areas of luminal compromise (Figure 2A-2B). There was severe calcific disease of the supra-celiac aorta (Figure 2C) and significant narrowing of the aortic bifurcation and common iliac arteries (Figure 2D).

Given her risk of bowel infarction and her poor quality of life, the patient chose to pursue treatment. Imaging of the thoracoabdominal aorta revealed severe exophytic calcified plaque, precluding direct aortic surgery. Stenting was also ruled out because her SMA was occluded at the ostium, with the disease extending well into the visceral aorta. Medical management with antiplatelet therapy (aspirin) and a high-intensity statin was already in place, but the progression of her symptoms necessitated intervention. Anticoagulation therapy was also considered essential for postoperative graft patency, given her small vessel size and marginal outflow. Due to her concurrent lower extremity claudication, an axillary-bifemoral bypass followed by a retrograde ilio-SMA bypass was considered. However, this option posed significant risks, as both her lower extremity and mesenteric perfusion would rely entirely on a single axillary artery. Between her left and right axillary arteries, the left demonstrated higher systolic pressure. Ultimately, the decision was made to prioritize her CMI and proceed with a left axillary-to-SMA bypass.

In the operating room, an 8mm polytetrafluoroethylene (PTFE) externally reinforced graft (W.L. Gore & Associates, Flagstaff, AZ) was utilized for the bypass. Control of the axillary artery was achieved, and the proximal anastomosis was performed in an end-to-side fashion. After restoring flow through the axillary artery, the graft exhibited a strong pulse. The graft was initially tunneled in a manner similar to an axillary-femoral bypass. Starting from the proximal axillary incision in the mid-axillary line, a subcutaneous tunnel was created inferior and posterior to the clavicle, with the graft coursing laterally until it pierced through the abdominal wall and entered the peritoneal cavity. A midline laparotomy was performed to access the abdomen. Within the peritoneal cavity, the graft coursed anterior to the left colon, beneath the transverse colon, and over the fourth portion of the duodenum. The graft was then anastomosed to the proximal segment of the anterior surface of the SMA in an end-to-side fashion, inferior to the transverse mesocolon. Following this, flow was successfully established to the SMA, with excellent pulses palpated in the mesenteric and antimesenteric borders of the small bowel, which appeared viable and demonstrated good peristalsis. The colon was unremarkable, with intact anatomy, no evidence of ischemia, and adequate blood flow confirmed by palpation and visual inspection. The retroperitoneum was reapproximated over the graft at the distal anastomosis.

**Figure 3:**
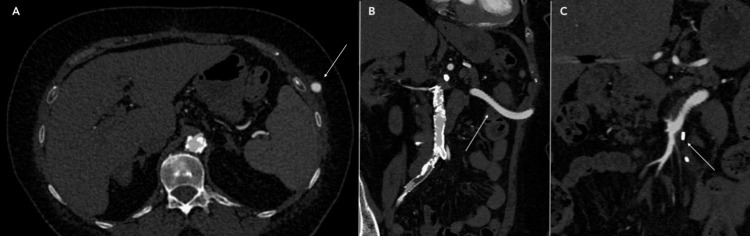
Post-operative computed tomography angiogram. The axillary graft (white arrow) is positioned in the traditional subcutaneous tunnel along the left flank (A) and enters the abdominal cavity through the left lateral abdominal wall (B) (white arrow). The anastomosis to the SMA and the distal SMA are widely patent (C) (white arrow).

Postoperative CTA demonstrated the axillary graft positioned in the traditional subcutaneous tunnel along the left flank (Figure 3A-3B) and entering the abdominal cavity. The anastomosis to the SMA and its distal branches were widely patent (Figure 3C).

The patient recovered without complications and was discharged on postoperative day (POD) 10, tolerating a regular diet. She was transferred to a skilled nursing facility for rehabilitation. At her follow-up appointment on POD 20, the patient demonstrated appropriate recovery, with resolution of postprandial abdominal pain and an improved appetite. CTA of the chest, abdomen, and pelvis performed during that visit revealed a widely patent bypass with excellent filling of the SMA and its distal branches. Long-term management included strict control of modifiable risk factors, continued anticoagulation therapy with rivaroxaban 2.5 mg twice daily, along with aspirin 81 mg, and routine imaging surveillance to ensure graft patency. She also remained on a high-intensity statin. The patient expressed high satisfaction with the outcome. A follow-up CT scan six months postoperatively confirmed the bypass remained widely patent, and the patient continued to deny any symptoms of mesenteric ischemia.

## Discussion

CMI remains a challenging clinical condition due to its progressive nature and significant risks, including acute mesenteric ischemia, bowel infarction, and death, necessitating timely intervention [6]. Traditional open approaches, such as anterograde aorto-mesenteric bypass or retrograde bypass using the infrarenal aorta or iliac arteries, have demonstrated excellent outcomes, with symptom improvement rates reported at 90 to 100% [3]. Endovascular techniques, including angioplasty and stenting, have emerged as the first-line treatment for patients with suitable lesions, offering high technical and clinical success rates [2,7]. However, these minimally invasive methods are associated with increased risks of reintervention and disease recurrence compared to open surgical procedures in patients with complex vascular anatomy or severe diffuse disease [8,9].

Patients with CMI often present with multiple comorbidities, diffuse vascular disease, and complex anatomy, all of which significantly elevate their mortality risk and complicate treatment decision-making. In such cases, open reconstruction leveraging the supraceliac aorta, infrarenal aorta, or iliac arteries as inflow sources remains a cornerstone of modern vascular surgery [10]. However, this approach was not viable for our patient due to severe calcific aortoiliac disease, which precluded direct aortic reconstruction. In these patients, the presence of severe aortic calcification often precludes the use of standard approaches, necessitating innovative solutions. Axillary-to-SMA bypass is one such solution that provides a robust alternative for mesenteric revascularization in cases where direct aortic reconstruction or endovascular therapy is contraindicated.

To our knowledge, four previous reports have demonstrated the feasibility of axillary-to-SMA bypass, particularly in the setting of acute mesenteric ischemia (AMI) [11-14]. For example, a prior study described the successful use of this technique to restore mesenteric perfusion in a patient with diffuse aortic disease, resulting in a patent graft and symptom resolution at 12 months [11]. In such cases, the graft can be tunneled deep to the pectoralis major and into the abdominal cavity under the costal margin, with omental coverage to reduce the risk of complications. While there is limited literature on the use of this approach for chronic mesenteric ischemia, our case highlights its effectiveness in achieving durable symptom relief and improving the quality of life in a complex clinical scenario. Importantly, our patient demonstrated complete resolution of her symptoms, restored appetite, and maintained long-term patency of the bypass graft. This outcome supports the axillary-to-SMA bypass as a versatile and underutilized option in the management of CMI.

## Conclusions

Advances in medical technology and increasing life expectancy have led to the growing prevalence of advanced atherosclerotic disease, necessitating innovative approaches to treatment. Axillo-mesenteric bypass grafting, although primarily reported for acute mesenteric ischemia, is emerging as a viable option for chronic disease, particularly in patients for whom traditional methods are contraindicated. This case demonstrates that axillary-to-SMA bypass can effectively restore mesenteric perfusion, alleviate symptoms, improve nutritional status, and enhance quality of life in patients with complex vascular anatomy. Future research should explore larger case series and long-term outcomes to better define the role of this approach in managing mesenteric ischemia.
